# Internet Addiction Among School Children: Cross-Sectional Analytical Study

**DOI:** 10.2196/68318

**Published:** 2026-03-26

**Authors:** Chamara Wijesinghe, Trivon Gunasekera, Piyumi Yashodhara, Asiri Rodrigo, Arunasalam Pathmeswaran, Lalith Kuruppuarachchi

**Affiliations:** 1 Department of Psychiatry, University of Kelaniya, 6 Thalagolla, Ragama, Sri Lanka, +94 0714818034; 2 Department of Public Health, University of Kelaniya, 6 Thalagolla Road, Ragama, 11010, Sri Lanka, +94 71 481 8034

**Keywords:** internet addiction, internet gaming disorder, World Wide Web, addiction, school children

## Abstract

**Background:**

Internet use is rapidly increasing in Sri Lanka. Excessive use can lead to addiction with significant consequences, particularly among adolescents. While internet addiction has been documented worldwide, data from Sri Lanka remain limited. A validated local tool is required to assess the prevalence and associated factors in this population.

**Objective:**

This study aimed to translate and validate the Young Internet Addiction Test (IAT) into Sinhala, assess the prevalence of internet addiction among school-going adolescents aged 15 to 19 years in the Western Province of Sri Lanka, and identify demographic and behavioral characteristics associated with internet addiction.

**Methods:**

We conducted a 2-phase cross-sectional analytical study in Colombo and Gampaha districts. Phase 1 involved translation and validation of the Sinhala IAT using confirmatory factor analysis (n=200) and test-retest reliability assessment (n=40). Phase 2 involved multistage stratified cluster sampling to recruit 2835 students. Participants completed self-administered questionnaires assessing demographics, internet use patterns, and internet addiction.

**Results:**

The Sinhala IAT demonstrated excellent internal consistency (Cronbach α=0.98) and strong test-retest reliability (*r*=0.95; *P*<.001). Among 2835 students with complete data, 1803 (63.6%) were current internet users. The overall prevalence of internet addiction among internet users was 12.6% (227/1803; 95% CI 11.2%‐14.0%), including mild addiction at 8.2% (147/1803; 95% CI 6.9%‐9.5%), moderate addiction at 3.5% (64/1803; 95% CI 2.7%‐4.5%), and severe addiction at 0.9% (16/1803; 95% CI 0.4%‐1.4%). No significant associations were found with sex (male and female; odds ratio 1.13, 95% CI 0.86-1.49; *P*=.14), age group (*P*=.23), or parental education (*P*=.34). The most common online activities were entertainment (1522/1803, 84.4%), gaming (1251/1803, 69.4%), and social media use (1127/1803, 62.5%). Mean daily use was 2.1 (SD 1.8) hours, with 10.0% (180/1803) reporting single sessions of ≥6 hours.

**Conclusions:**

This study provides the first systematic evidence of internet addiction in adolescents in Sri Lanka. The predominance of mild to moderate severity suggests an opportunity for early intervention.

## Introduction

The rapid expansion of digital technologies and internet access has transformed communication, education, and social interaction worldwide [1-3]. Over the past 2 decades, the internet has evolved from a specialized communication network into an integral part of daily life, influencing learning, entertainment, and social relationships. Adolescents constitute one of the fastest-growing groups of internet users, particularly in low- and middle-income countries, where access has expanded rapidly with the widespread availability of smartphones and mobile broadband services, and according to estimates, there are almost 3 billion users worldwide, with adolescents among the most active users [4-7]. While internet use offers substantial educational, informational, and social benefits, concerns have been raised regarding excessive and uncontrolled patterns of use. Harmful or problematic internet use has been associated with a range of adverse outcomes, including sleep disturbance; reduced academic performance; impaired social functioning; and mental health problems, such as depression, anxiety, and suicidal ideation [8-11]. These concerns have led to increasing attention from clinicians, educators, and public health professionals.

Internet addiction was first described by Young [9] as a behavioral condition. Internet addiction is characterized by impaired control over internet use, preoccupation, tolerance, withdrawal-like symptoms, and functional impairment [9,12-14]. Subsequent research has supported the clinical relevance of this construct, with growing consensus that certain forms of excessive internet use share features with other behavioral addictions [13,15,16]. Although internet addiction is not uniformly classified across diagnostic systems, related conditions, such as internet gaming disorder, have been recognized as conditions warranting further study [15].

The Young Internet Addiction Test (IAT) [14] is the most extensively used study tool to assess internet addiction and has been validated for use in many countries including India [17-24]. Many studies throughout the world have shown that internet addiction is becoming a bigger problem, especially for young people. Earlier studies conducted in Western populations reported wide variability in the prevalence of internet addiction. For example, Fortson et al [25] found that among 411 American undergraduate students, 50% met the criteria for internet abuse and 25% for internet dependence. Similarly, Cash et al [26] reported prevalence rates ranging from 1.5% to 8.2% among students in the United States and Europe. In Italy, Poli and Agrimi [27] reported that 0.8% and 5% of students had severe and moderate levels of internet addiction, respectively.

This pattern is not unique to Western countries, and several studies have also been conducted in Asia. Recent systematic reviews estimate the global prevalence of internet addiction among adolescents and young adults to range between 7% and 15%, with higher rates reported in Asian countries compared with Europe and North America [28]. Similar findings have been observed in Hong Kong and Taiwan, where internet addiction is often linked to gaming and social media use [29,30]. More recent studies from South Korea (2021) and Japan (2022) report rates of 14.2% and 8.7%, respectively, among adolescent populations [31,32].

Several studies on internet addiction have been conducted in the Indian subcontinent. Goel et al [31] identified a low prevalence of internet addiction, with only 0.7% of the sample of college students having features of internet addiction. This varied considerably, with a study conducted by Gedam et al [32] reporting a total prevalence of internet addiction of 19.85% in a student population in India [33]. An even higher prevalence was found in a study conducted by Subashini and Praveen [33], with 57.7% of sampled college students having features of internet addiction.

In Sri Lanka, internet penetration has increased steadily over the past decade, particularly among school-aged populations, in parallel with broader national gains in information and communication technology infrastructure [34-36]. Despite this rapid growth, local evidence on the prevalence and correlates of internet addiction among school children remains limited. Chandradasa and Rodrigo [37] reported the presence of internet gaming disorder among adolescents, highlighting the significance of internet-related behavioral problems in the local context [38]. This leaves a gap in understanding the broader patterns of internet addiction among adolescents. Moreover, no validated Sinhala language assessment tools exist, baseline prevalence data are unavailable, and risk factors specific to Sri Lankan adolescents remain unknown.

This study addresses these gaps by translating and validating the Young IAT for Sinhala-speaking populations. The study aimed to estimate the prevalence of internet addiction among school children in the Western Province of Sri Lanka and to examine factors associated with problematic internet use. A clearer understanding of these patterns is essential to inform school-based mental health promotion, preventive strategies, and policy development within the education sector.

## Methods

### Overview

We conducted a school-based cross-sectional analytical study in the Colombo and Gampaha districts of Sri Lanka. The cross-sectional design was chosen as the most appropriate for several reasons. This is the first systematic study of internet addiction within a large adolescent population in Sri Lanka. While this design provides essential baseline data for future longitudinal and interventional studies, it cannot establish causality. In comparison to other districts, Colombo and Gampaha were selected for several reasons: (1) the highest population density in Sri Lanka, providing efficient access to a large sample; (2) the greatest internet penetration rates in the country (>70%), ensuring adequate numbers of internet users; (3) diversity of urban and semiurban areas; (4) a range of school types (government, private, and international) and socioeconomic backgrounds; and (5) representativeness of broader trends in internet adoption and use patterns among Sri Lankan adolescents. The cross-sectional design enables understanding of multiple potential correlates concurrently.

Our primary objectives were to evaluate the prevalence of internet addiction in a Sri Lankan student population and determine personal characteristics associated with internet addiction, including age, sex (male and female), language, and year of study. We explored patterns of internet use, such as social media, gaming, and web surfing, associated with internet addiction.

### Study Population

Students of both sexes were eligible for inclusion if they were aged between 15 and 19 years and studying in grades 10, 11, 12, and 13 in the Gampaha and Colombo districts of Sri Lanka. This age group was selected because adolescence is a critical developmental stage characterized by high internet use and vulnerability to behavioral addictions. As the study involved validation of the Sinhala translation of the IAT, students without adequate Sinhala proficiency were excluded. This constitutes a considerable limitation as exclusively Tamil-speaking students were not included in the study.

### Sampling Methods and Sampling Strategy

A multistage stratified cluster sampling method was selected. A classroom was considered a cluster. Considering the diversity of the school population, the sample was stratified at 2 stages. First, the sample (clusters) was divided equally into 2 strata according to school grades. The strata were defined as grades 10 and 11 (Ordinary Level classes) and grades 12 and 13 (Advanced Level classes). Within each of these strata, the sample was divided to include students from government, private, and international schools. Students from government schools were stratified according to the functional classification of schools adopted by the Ministry of Education, namely, types 1AB, 1C, and type 2 [39].

Schools within each district were organized according to the type of school, and the number of students to be sampled from each grade was determined using probability proportional to size based on the student population in the specific grades in the different types of schools (government—1AB, 1C, and type 2; private; and international). To enhance the distribution of the sample over the district, only one class from Ordinary Level classes and one from each type of Advanced Level classes were randomly selected from a single school.

### Sample Size Calculation

The required sample size was calculated using the following formula: sample size (N) = *Z*^2^ × *p*(1–*p*)/*d*^2^, where *Z* is the value corresponding to the CI (1.96 for 95%), *p* is the anticipated population proportion (*p*=0.1), and *d* is the absolute precision (*d*=0.025).

On the basis of the literature, 4% to 10% of users have internet addiction [27]. Studies in the general population and adolescents report a lower prevalence of internet addiction, ranging from 0.3% to 0.07% [32]. To ensure a conservative and sufficient sample size, a prevalence of 10% of internet users was taken as the cutoff. With a 95% CI and a margin of error set at 2.5%, the minimum number of positive cases required was calculated as 554 as follows:

Sample size (n)=*Z*^2^ × *p*(1–*p*)/*d*^2^

n=(1.96)^2^ × 0.10(0.90)/(0.025)^2^

n=3.84 × 0.09/0.000625

n=553.9, or approximately 554 internet users with addiction.

As of July 2014, approximately 19.9% of the Sri Lankan population was estimated to be internet users having access to the internet at home via any type of device (computer or mobile) [34,35]. Therefore, to obtain a sample of 139 adolescents with frequent internet use (assuming 20% of the student sample are frequent users), a total of 2800 students were targeted. Our study sample comprised 2835 students. Nonresponse was minimal; incomplete questionnaires were excluded from the final analysis.

Thereafter, the study was divided into 2 phases to allow systematic data collection: initial validation of the translated Sinhala version of the IAT (phase 1) and subsequent data collection to evaluate the prevalence of internet addiction (phase 2).

### Phase 1: Validation of the Translated Sinhala Version of the IAT

It was necessary to ensure that the tool was validated before being used to measure internet addiction. Previous validations of the IAT have offered several models, ranging from 1 factor to 6 factors [32-35,37-39]. Recent studies have identified that a 1-factor or 2-factor model can be used effectively in validating the IAT and have shown no clear benefit over one another [23]. With the above evidence, we proceeded with a single-factor validation model, adopting a unidimensional approach.

On the basis of the guidelines proposed by George and Mallery [39] for interpreting Cronbach α values [40,41], internal consistency was interpreted using the criteria shown in Table 1.

**Table 1.** Interpretation of Cronbach α coefficient.

Cronbach α score	Degree of internal consistency
>0.9	Excellent
0.8‐0.9	Good
0.7‐0.8	Acceptable
0.6‐0.7	Questionable
0.5‐0.6	Poor
<0.5	Unacceptable

To measure test-retest reliability, the same test was given to the same students on 2 occasions and the scores were correlated using Pearson correlation coefficient. The score obtained the first time the test was given was T1, and the score obtained the second time was T2. The scores on the 2 occasions were then correlated. This correlation was known as the test-retest reliability coefficient, or the coefficient of stability.

The closer each respondent’s scores were on T1 and T2, the more reliable the test measure. A stability coefficient of 1 would indicate that each respondent’s scores were perfectly correlated; that is, each respondent scored the exact same value on T1 as they did on T2. A correlation coefficient of 0 indicates that the respondents’ scores at T1 were completely unrelated to their scores at T2; therefore, the test was not reliable.

Correlation is an effect size, and the strength of the correlation between items can be described using the interpretation guide for the absolute value of the correlation coefficient (Table 2).

**Table 2.** Interpretation of coefficients of stability for test-retest validity of a scale.

Coefficient of stability	Interpretation
≥0.9	Excellent reliability
0.8‐0.9	Good reliability
0.7‐0.8	Acceptable reliability
0.6‐0.7	Questionable reliability
0.5‐0.6	Poor reliability
<0.5	Unacceptable reliability

In our study, we used a 1-factor model assessing the Cronbach α score for the 20 items on the translated IAT. A statistical expert calculated the ideal sample size for validation before data collection. According to the widely accepted participant to item ratio approach, each item should include 2 to 20 participants, and a minimum sample size of 100 to 250 participants is required for validation [42]. A sample size of 200 individuals (10 participants per item) was considered adequate because the IAT has 20 items. To assess test-retest validity, 20% (n=40) of the sample was also enlisted.

The first 200 students who identified as internet users were included in validation analysis. This was done by calculating the Cronbach α coefficient. A score of ≥0.7 indicated that the interview schedule in Sinhala had a high level of validity and internal consistency.

For cross-cultural adaptation of psychometric instruments, the original IAT was translated into Sinhala by a nonmedical professional competent in both Sinhala and English. This was subsequently reviewed by a consultant psychiatrist and a specialist in computer technology. Discrepancies were discussed and resolved to create a single preliminary Sinhala version. They identified a few items requiring cultural modification to ensure relevance and comprehensibility: (1) modification to age-appropriate language, (2) adjustment of phrasing for natural Sinhala expression while preserving meaning, and (3) clarification to reflect Sri Lankan social context.

It was then retranslated into English by a different nonmedical professional, and the retranslated version was cross-checked by the review team. Necessary corrections and amendments were made to the translated Sinhala version so that the retranslated English version was as close as possible in meaning to the original interview schedule.

### Phase 2: Data Collection and Assessment of the Prevalence of Internet Addiction

Following the abovementioned validation, the study tool was used to assess the presence and severity of internet addiction in the sample population. Internet addiction was assessed using the Sinhala translated and validated version of the IAT. A questionnaire assessing demographic data and the IAT was administered concurrently to the study population. To ensure the questionnaire was comprehensible across varying literacy levels, the language was simplified, and a research assistant was present during administration to provide clarifications when needed.

### Data Management and Statistical Analysis

All gathered data were handled confidentially, with each participant assigned a unique reference number to ensure anonymity. SPSS software (version 23.0; IBM Corp) was used for data entry and analysis.

The translated IAT was validated using the first 200 entries; test-retest reliability was evaluated using the Pearson correlation coefficient, and internal consistency was evaluated using Cronbach α.

Following validation, the entire dataset was analyzed to assess the level of internet addiction and its nature. Descriptive statistics (means, SDs, frequencies, and percentages) were used to summarize sociodemographic variables and internet use patterns. Chi-square tests were used for categorical variable comparisons (sex [male and female] and academic profile), whereas independent 2-tailed *t* tests analyzed continuous variables (age differences between groups). One-way ANOVA was applied for multigroup comparisons, and the Pearson correlation coefficient was used to assess relationships between continuous measures.

Prior to statistical analysis, the dataset was screened for missing values. Missing data were analyzed for patterns using the Little MCAR (Missing Completely At Random) test to determine whether data were missing completely at random. For variables with less than 5% missing data, listwise deletion was used.

### Ethical Considerations

Ethics approval and other necessary permissions were obtained prior to the study. Ethical clearance for this study was obtained from the ethical clearance committee of the Faculty of Medicine, Ragama, University of Kelaniya (P/240/12/2014). Formal permission to translate and validate the IAT into Sinhala was obtained from its author, Dr Kimberley Young. All participants received an information sheet outlining the study details and what was required of them. Parental notification was conducted through school principals, who were asked to inform parents about the study and communicate any objections to their children’s participation. No parental objections or complaints were received, and students provided written assent after being informed about voluntary participation and confidentiality measures. In addition, written approval was granted by the relevant school principals to conduct the study in their respective schools, and informed written consent was obtained from students aged 18 years and above for participating in the study. No compensation was provided to participants, and participation was voluntary.

The data collected were anonymized and stored in a password-protected electronic database accessible only to the principal investigator and authorized research staff, and all paper-based records were locked in secure storage.

### Problems Anticipated and Measures Taken to Minimize Them

We anticipated that students might not provide fully accurate information regarding their internet use due to fear of reprimand or other concerns. An anonymous self-administered questionnaire was administered under the supervision of a researcher. Subsequently, students were instructed to place the completed questionnaires into a closed collection box to ensure that their responses could not be traced to them or seen by other students. Furthermore, responses on internet use duration were compared with other reported digital behaviors, such as time spent on social media, gaming, and educational browsing. Extreme or inconsistent responses were flagged and excluded during data cleaning. Nonresponse and incomplete questionnaires were excluded from the final analysis.

## Results

A total of 200 students participated in the validation phase. The Sinhala IAT demonstrated excellent internal consistency with a Cronbach α of 0.98, and individual item analyses confirmed high interitem correlations (all >0.70). Test-retest reliability, assessed after a 2-week interval in a subsample of 40 students, showed a Pearson correlation coefficient of 0.95 (*P*<.001), indicating strong temporal stability (Table 3 and Table 4).

**Table 3.** Factors associated with internet addiction (n=1803 internet users).

Variable	Addiction present	Addiction absent	Odds ratio (95% CI)	*P* value	Effect size
Sex, n (%)			1.13 (0.86‐1.49)	.14	0.03
Male	126 (12.9)	852 (87.1)			
Female	101 (12.3)	721 (87.7)			
Age group (y), n (%)			—^a^	.23	0.05
15‐16	105 (15.4)	577 (86.3)			
17‐18	106 (11.0)	855 (88.3)			
19	16 (10.0)	144 (88.2)			
Academic level, n (%)			1.10 (0.83‐1.46)	.09	0.06
Ordinary level (grade 10‐11)	117 (14.1)	712 (85.9)			
Advanced level (grade 12‐13)	110 (11.3)	861 (88.7)			
Primary activities^b^, n/N (%)					
Gaming			0.98 (0.74‐1.30)	.83	0.01
Yes	156/227 (12.5)	1092/1573 (87.5)			
No	71/227 (12.9)	481/1573 (87.1)			
Social media			0.96 (0.72‐1.27)	.69	0.01
Yes	139/227 (12.4)	985/1573 (87.6)			
No	88/227 (13.0)	588/1573 (87.0)			

a Not applicable.

b Primary activity percentages were calculated within addiction groups. Internet addiction was defined as an Internet Addiction Test score of ≥31. Of 1803 current internet users, 227 (12.6%) met the criteria for internet addiction. Data on primary activities were missing for 0.2% (3/1576) of the participants in the addiction-absent group; therefore, percentages for these variables were calculated using available data (n=1573).

c All effect size values represent Cramer V.

**Table 4.** Validation results of the Sinhala Internet Addiction Test.

Psychometric property	Result	Interpretation
Internal consistency		
Cronbach α	0.98	Excellent
Item-total correlation (range)	0.72-0.90	All items contribute strongly
Test-retest reliability		
Pearson correlation (n=40^a^; 95% CI)	0.95^b^ (0.91-0.97)	Excellent stability
Time interval	2 wk	—^c^

a The number of participants who completed the test-retest reliability assessment (ie, they took the Sinhala Internet Addiction Test twice, 2 weeks apart, to measure the temporal stability of the test).

b *P*<.001.

c Not applicable.

**Figure 1.**
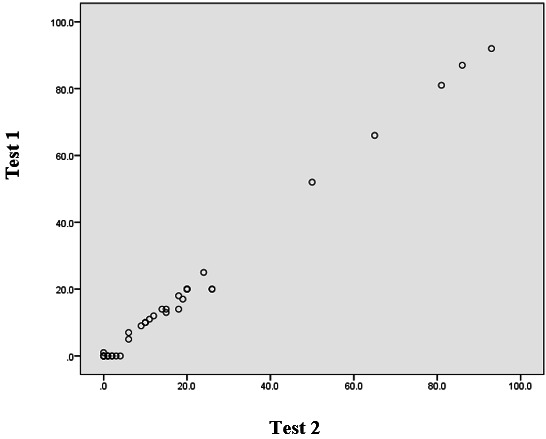
Correlation between the test (test 1) and retest (test 2) scores.

This high correlation coefficient indicated that the Sinhala translation maintained consistent measurement properties over time (Figure 1). These validation results confirmed that the Sinhala version of Young IAT possessed both high internal consistency and excellent test-retest reliability, making it suitable for use in the subsequent prevalence study.

On the basis of the scale of the internet addiction questionnaire, internet addiction score ranged from 2 to 97, with a mean score of 17.4 (SD 23.8). Among the total sample, 8.0% (227/2835) showed features of internet addiction. Among internet users specifically, the prevalence was 12.6% (227/1803). The severity of the addiction was categorized as mild, moderate, and severe. The results revealed a mild addiction (147/2835, 5.2% of the total sample; 147/1803, 8.2% of internet users) in the majority, followed by moderate addiction (64/2835, 2.3% of the total sample; 64/1803, 3.5% of internet users). However, just under 1% (16/1803, 0.9%) of internet users showed evidence of severe addiction (Table 5 and Table 6).

**Table 5.** Sample characteristics and internet use patterns.

Characteristic	Values
Demographics	
Age (y), mean (SD)	16.7 (1.2)
Sex (n=2835), n (%)	
Female	1344 (47.4)
Male	1491 (52.6)
Academic level (n=2387), n (%)	
Ordinary level (grade 10-11)	1108 (46.4)
Advanced level (grade 12-13)	1279 (53.6)
Language (n=2387), n (%)	
Sinhala	2323 (97.3)
English	64 (2.7)
Internet use	
Current internet users (n=2387), n (%)	
Yes	1803 (75.5)
No	584 (24.5)
Among internet users (n=1803)	
Daily use duration (h), mean (SD)	2.1 (1.8)
Session of ≥6 h, n (%)	181 (10.0)
Primary devices (n=1803), n (%)	
Laptop	1408 (78.1)
Mobile phone	1273 (70.6)
Tablet	200 (11.1)
Primary location (n=1803), n (%)	
Home	1473 (81.7)
Friend’s house	449 (24.9)
Internet café	258 (14.3)
Top activities (n=1803), n (%)	
Entertainment	1522 (84.4)
Email	1307 (72.5)
Gaming	1251 (69.4)
Social media	1126 (62.5)
Schoolwork	1081 (60.0)
Pornography	371 (20.6)

**Table 6.** Prevalence of internet addiction by severity.

Severity category	IAT^a^ score	Internet users (n=1803), n (%)	95% CI	Total sample (n=2835), n (%)
No addiction	≤30	1576 (87.4)	85.9‐88.8	1872 (66.0)
Any addiction	≥31	227 (12.6)	11.2‐14.0	270 (9.5)
Mild	31‐49	147 (8.2)	6.9‐9.5	175 (6.2)
Moderate	50‐79	64 (3.5)	2.7‐4.5	76 (2.7)
Severe	≥80	16 (0.9)	0.4‐1.4	19 (0.7)

a IAT: Internet Addiction Test.

## Discussion

### Principal Findings

This study demonstrates that internet addiction is prevalent among school children in the Western Province of Sri Lanka, with a substantial proportion exhibiting mild to moderate levels of problematic use. These findings add to the limited body of evidence from Sri Lanka on adolescent internet addiction and highlight that problematic use is not confined to university or clinical populations. The prevalence pattern observed in this study is broadly consistent with findings from other South Asian countries, including India and Bangladesh, where studies among adolescents have reported a wide range of prevalence estimates depending on methodology, age group, and assessment tools used [23,24,32]. Differences in reported prevalence across studies may reflect variations in internet access, cultural norms, parental supervision, and educational demands.

The predominance of mild and moderate addiction observed in this study mirrors trends reported globally. Meta-analyses and large-scale studies from China, Europe, and other regions suggest that early-stage problematic internet use is considerably more common than severe addiction among adolescents [28,43]. This pattern is important from a public health perspective, as it suggests opportunities for early identification and intervention before more severe impairment develops. The lack of significant associations between internet addiction and basic sociodemographic characteristics in this study is consistent with findings from Hong Kong, Taiwan, and South Korea [29,30,44]. Instead, behavioral factors related to patterns of use appear to be more salient. Longer duration of use and recreational or gaming-focused internet activities have been repeatedly associated with higher addiction scores across different cultural settings [15,25,38]. These findings support the view that how the internet is used may be more important than who is using it.

From a public health and educational perspective, the findings underscore the need for school-based approaches to promote healthy internet use. International evidence suggests that digital literacy education, parental engagement, and clear school policies regarding internet use can reduce harmful patterns and associated psychosocial problems [27,45]. In Sri Lanka, where internet access among school children continues to expand rapidly, integrating such interventions into existing school health and education programs may be both feasible and effective.

### Limitations

While the cross-sectional study design provides a snapshot at one point in time and helps estimate prevalence and associated factors, it does not allow for the establishment of causal relationships. Therefore, future longitudinal studies are needed for more comprehensive assessment.

Colombo and Gampaha districts have the highest internet penetration, the greatest urbanization, and higher average socioeconomic status. So the prevalence, patterns, and correlates of internet addiction likely differ substantially compared with rural areas with limited connectivity, which represents a major limitation. Our findings should not be extrapolated to these contexts without appropriate research.

As the study involved validating the Sinhala translation of the Young IAT, the Tamil-speaking population was not assessed for internet addiction. Therefore, these findings may not be generalizable to the entire Western Province and limit generalizability across ethnic groups. Tamil-speaking communities may have different cultural attitudes toward technology use, different parental supervision patterns, different media consumption preferences, and potentially different prevalence of problematic use. There is potential for selection bias, as the findings may not reflect the experiences of adolescents in rural and remote areas, and the exclusion of Tamil-speaking students may introduce ethnic and linguistic bias.

The prevalence of internet addiction among internet users in the study sample was 12.6% (227/1803), and only 0.9% (16/1803) were identified as having severe internet addiction. This is less than that identified in some studies in other countries, including India [24,32,33]. This may have been due to the students not being forthcoming with information when data were collected in a school setting by research assistants, even though the questionnaire was self-administered and confidential. Students may have underreported problematic use, creating the desirability bias. Therefore, many studies in this area have been conducted using internet-based surveys, which are more anonymous and may yield more accurate information, potentially minimizing desirability bias.

We did not collect detailed socioeconomic status indicators, such as family income, parental occupation, household assets, or parental education level. This represents a significant limitation, as socioeconomic status likely operates through multiple pathways to influence internet addiction risk. We did not screen for depression, anxiety disorders, attention-deficit/hyperactivity disorder, conduct disorders, or other psychiatric conditions that commonly occur with internet addiction. Future research must include comprehensive mental health assessment as well.

### Conclusions and Future Recommendations

Internet addiction is prevalent among school children in the Western Province of Sri Lanka, predominantly at mild to moderate levels. Patterns of internet use, rather than sociodemographic characteristics, are key correlates. Incorporating screening and preventive interventions into school health and education programs may help mitigate the long-term psychological and social consequences of excessive internet use among adolescents.

## Abbreviations

IAT: Internet Addiction Test
MCAR: Missing Completely At Random
